# A Training Intervention through a 360° Multisource Feedback Model

**DOI:** 10.3390/ijerph18179137

**Published:** 2021-08-30

**Authors:** Elena Sureda, Salvador Chacón-Moscoso, Susana Sanduvete-Chaves, Albert Sesé

**Affiliations:** 1 Department of Psychology, University of Balearic Islands, 07122 Palma, Spain; elena.sureda@uib.es; 2 Experimental Psychology Department, Universidad de Sevilla, 41018 Sevilla, Spain; sussancha@us.es; 3 Department of Psychology, Universidad Autónoma de Chile, Santiago 7500138, Chile; 4 Balearic Islands Health Research Institute (IdISBa), 07120 Palma, Spain

**Keywords:** healthcare performance, professional development, postgraduate training, 360° multisource feedback model, evaluation culture

## Abstract

Physicians and other health sciences professionals need continuous training, not only in technical aspects of their activity but also in nontechnical, transversal competencies with a cost-efficient impact on the proper functioning of healthcare. The objective of this paper is to analyze the behavioral change among health professionals at a large public hospital following a training intervention on a set of core nontechnical competencies: Teamwork, Adaptability-Flexibility, Commitment-Engagement, Results Orientation, and Leadership Skills for Supervisors. The 360° Multisource Feedback (MSF) model was applied using three sources of information: supervisors, co-workers, and the workers themselves (self-assessment). A quasi-experimental pretest–post-test single-group design with two points in time was utilized. The training intervention improved the scores of only one of the trained competencies—the “Results Orientation” competency—although the scores were slightly inflated. Moreover, significant discrepancies were detected between the three sources, with supervisors awarding the highest scores. The magnitude of behavioral change was related to certain sociodemographic and organizational variables. The study was not immune to the ceiling effect, despite control measures aimed at avoiding it. The empirical evidence suggests that the 360° MSF model must be maintained over time to enhance and reinforce an evaluation culture for better patient care.

## 1. Introduction

In the health sector, competence assessments are critical to enhancing training curriculum, hiring, certification and recertification, incentives, and promotions. The literature makes reference to different tools to carry out the competence assessment, but the 360° Multisource Feedback (MSF) model is considered the most appropriate in terms of cost and time [1], as well as reliability and validity [2,3]. The 360° MSF model draws on several different sources all familiar with the role in question to evaluate the performance of professionals. MSF models usually include questionnaires, including self-assessments by the evaluated professionals, evaluations by their superior/s, peers in their professional category or/and other categories, and subordinates or patients. All assessments are then compared to obtain an overall score on the professional’s performance.

Although the MSF model was initially developed in the corporate world and can be multidisciplinary, it has been used mainly to evaluate physicians in a healthcare setting, with a smaller number of studies on other healthcare professionals. At health organizations, MSF has been mainly used to analyze service quality and detect opportunities for improvement, so it usually assesses practicing professionals and analyzes nuclear or transversal competencies (related to nontechnical aspects of the profession) [4,5,6,7,8,9,10,11,12,13,14,15,16,17]. According to the evidence, the multisource evaluation more efficiently evaluates aspects related to communication, professionalism, management, and interpersonal relationships [2,18,19,20].

However, the MSF is not an adequate method for assessing a professional’s specific or technical competencies. This is mainly because co-workers, who often have no direct observation of job performance and rely on secondary information, are unable to provide a comprehensive assessment of all relevant job skills [21]. Another problem is the inherent difficulty of establishing a manageable number of items to measure the use of technical job skills [7].

The most important elements of the 360° MSF model reviewed in the literature are the following [22]. First, the professional to be evaluated must participate in the selection process of raters [23]. Second, the most important criterion is selecting raters who have direct information and knowledge about the person to be assessed. Third, the behaviors and outcomes to be assessed must be clearly stated. Fourth, an appropriate sample size of raters for each source should be used in order to increase accuracy and reliability. Fifth, all raters should use a single Likert-type scale for their assessment to facilitate comparisons and consensus. Sixth, feedback and collective evaluation processes ought to be developed and given very respectfully, and seventh, a log should be kept of recommendations and improvements over time.

Based on its main characteristics, the MSF model differs from others with respect to the number of sources and raters who provide the feedback [24]. Thus, triangulating three or more different sources of evidence can allow the strengths of each individual source to compensate for weaknesses on the part of other sources, thereby contributing to a more accurate assessment than one based on any single source [25]. In terms of the quantity of raters, a multisource evaluation needs to be valid and reliable; Donnon et al. [2] recommend a minimum of eight co-workers and 25 patients. Overeem et al. [26] suggest that five co-workers and 11 patients are required, while Berk [27] proposes five nurses, four patients, and three colleagues. In any case, the most important condition for the evaluators is that they have enough information to evaluate the real behavior of the professional in question [27]. This should be a fundamental selection criterion for potential raters.

Some authors have argued that in order for a multisource evaluation to be successful, the quality feedback it produces must be used to design an intervention plan for improving performance. This, the authors argue, most faithfully represents the original intent of the 360° MSF model [22,28]. Most of the existing studies apply transversal designs, preventing an analysis of the sensitivity to change after an intervention or an assessment of the factors that influence an intervention’s effectiveness. A few studies, such as the Physician Assessment Review (PAR), the Sheffield Peer Review Assessment Tool (SPRAT) [28,29,30,31], or some developed for assessing nursing students [19], or anesthesia residents [20], present a longitudinal design, but in the best of cases, only the intention to change is analyzed [9,32,33,34,35,36,37,38]. Even studies that have reported improved performance over time recognize that the behavioral change produced may not be entirely attributable to the feedback provided [29].

To contribute more empirical evidence and address this lack of longitudinal MSF studies in the literature, the main objective of this study is to analyze potential behavioral changes in different health professionals belonging to a public hospital following a training intervention in four core competencies: Teamwork, Adaptability-Flexibility, Commitment-Engagement, and Results Orientation. In a subsample of team leaders, a fifth competency, Leadership Skills, was also included. All skills were evaluated using the 360° MSF model and a longitudinal pretest–post-test design. A second objective was to analyze any discrepancies between the sources of information considered, as well as their relationship with sociodemographic and organizational variables (job satisfaction and burnout).

## 2. Materials and Methods

### 2.1. Participants

A panel of experts in four hospital areas (medical, surgical, laboratory, and management), with the full involvement of the management team of the hospital, established the sampling criteria with the permission of the government health system. One service was chosen for each of four hospital areas with similar organizational structures; a decision was made to include all professional categories with a representative number of workers. A total of 127 potential participants were selected once the criteria of representativeness and inclusion/exclusion of the 360° MSF model were applied. As participation was voluntary in accordance with the principles of the MSF model, 94 of the original 127 opted to participate in the study, that is, 74.01% of the total selected; all completed the training program and the protocols in the pretest and post-test phases.

### 2.2. Procedure

A quasi-experimental pretest–post-test design was implemented to evaluate a set of nuclear competencies in two stages. Participants completed a training program (intervention) that aimed to improve their performance in these competencies. Both the pre-post measures and the intervention were interspersed as part of their routine professional practice. The time interval considered between the two measures was approximately one year [39,40].

In this context, the 360° MSF model consisted of nine phases developed over three years (2016–2018) (Table 1). A panel of experts identified a subset of four core competencies associated with strategic hospital management: Teamwork (9 items), Adaptability-Flexibility (5 items), Commitment-Engagement (7 items), and Results Orientation (8 items); a fifth subset, Leadership Skills (10 items), was also included but exclusively for team leaders. Competencies were disaggregated into behavioral indicators that were assigned items on the questionnaire. A 10-point Likert-type scale was divided into five levels for better interpretation (not developed 0–2, subpar 2–4, adequate 4–6, advanced 6–8, and expert 8–10).

Prior to the evaluation, an analysis of work interactions between the professionals was carried out to prepare the circuits. This way, the aim was to avoid biases related to personal preferences and to select raters who had actually observed the performance of the participant in question. Three types of raters were established for each participant. Participants who held a leadership role, for example, had to complete the questionnaires about their subordinates (team) and co-workers (other positions of responsibility), plus their self-assessment. People holding intermediate positions of responsibility were evaluated by their immediate superiors, by other heads, and by members of their team. The remaining participants were evaluated by their immediate superior and their co-workers, besides completing their self-assessment. As a general rule, an average of six co-workers was randomly chosen based on the interaction analysis.

The training intervention involved workshops in which the selected core competencies were analyzed and trained: supervisors completed a 5-hour module and personnel completed two 3-hour modules. Supervisors received information and training on the necessary skills and resources to complete feedback interviews and deal with potential biases.

After the training intervention was completed and enough time elapsed to assess the transfer of knowledge associated with the training, the supervisor’s direct assessment (or the collective assessment in the case of more than one), the average of the co-workers’ scores, and the self-assessment were collected for each item. Protocols whose scores were either the minimum or the maximum (0 or 10 points) without any variability were discarded to avoid floor and ceiling effects. The overall score for each competency was obtained by calculating the average of the scores for all items.

**Figure 1:**
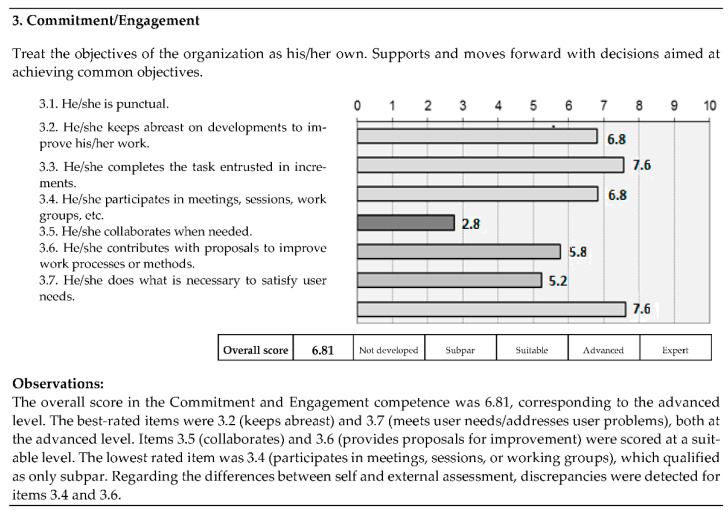
Example of an individual feedback report (Commitment-Engagement competency).

Participants received the feedback of the 360° MSF model in carefully drafted personalized reports presenting the final score of each item, the overall score of each competency, and the level reached. The reports also included observations on the participant’s results and noted any discrepancies between the sources (Figure 1). Comparative data were also offered with respect to the average scores in their professional category and service/area. In the re-evaluation, a pretest–post-test comparative chart was provided to highlight any improvements in the trained competencies. An assessment was considered discrepant when the mean of the external rater scores differed from the self-assessment scores by more than two points (on the scale of 0–10) and for more than 50% of the items. In those cases, the supervisor and the subordinate held a meeting to reach an agreement on a final score for the discrepant items.

The protocol also included a set of sociodemographic variables: age, sex, seniority with the company, area (medical, surgical, laboratory, or management), profession (supervisor, physician, nurse, technical specialist, nursing assistant, or administrative staff), types of contracts (permanent or temporary), official patient complaints received by each service and attributable to the organization, the professional’s aptitude, and/or the professional’s attitude. Finally, psychometric measures of job satisfaction (the Job Satisfaction Questionnaire) [41] and Burnout Syndrome (the Maslach Burnout Inventory) [42] were included to assess the effect these could have on behavioral changes in job performance.

### 2.3. Statistical Analysis

To study the effect of the training intervention on the selected competencies as well as any bias between the three feedback sources, a mixed ANOVA was carried out with two factors (2 × 3). As part of this design, factor A (within subjects) operationalizes the final score of each participant in the pretest and post-test phases, while factor B (between subjects) represents the three feedback sources (supervisors, co-workers, and self). With this analytical configuration, it is possible to determine if the training intervention yields improvement (factor A), and whether there are discrepancies between the scores of the three sources, by estimating simple effects tests of B on A. The observed power as a function of the sample size for the ANOVA was 0.84. Tests of mean comparisons for related samples were also applied in order to analyze the effect of the training program for each of the items, and the profession of participants was also considered as an independent variable. The Kolmogorov–Smirnov normality test and the 95% confidence intervals of skewness (g1) and kurtosis (g2) Bliss indices were estimated for the scores of all competencies. Finally, the magnitude of the competency change was compared with the sociodemographic and organizational variables by means of independent t-tests with categorical variables and Pearson correlation coefficient (*r*) for continuous variables. All analyses were carried out using the statistical program SPSS 25.0 [43].

## 3. Results

Supervisors represented 13.83% of the participants; 53.8% of the supervisors were men, with an average age of 56.62 (*SD =* 5.98), seniority of 27.46 years (*SD =* 7.76) with the company. All had permanent contracts. Regarding the staff (86.17%), 27.16% were physicians, 27.16% nurses, 23.46% nursing assistants, 9.88% laboratory technicians and 12.34% administrative staff; 74.1% were women and the average age was 41.84 (*SD =* 9.99), the mean of seniority in the company was 14.26 years (*SD =* 10.15); 53.1% had a temporary contract vs. 46.9% with a permanent one. Finally, by areas, 44.44% belonged to the medical service, 24.69% to surgery, 20.99% to laboratories, and 10% to administrative services.

The Kolmogorov–Smirnov tests and 95% CI skewness and kurtosis Bliss indices for global ratings of competencies showed a normal distribution for “Adaptability-Flexibility”, “Results Orientation”, and “Leadership Skills” at T1, while “Teamwork” was negatively skewed and platykurtic, and “Commitment-Engagement” was also negatively skewed but mesokurtic. At T2, only “Leadership Skills” retained a normal distribution, while “Teamwork”, “Adaptability-Flexibility”, and “Results Orientation” skewed negative with a leptokurtic distribution; finally, “Commitment-Engagement” also skewed negative and was mesokurtic (Table 2).

The best-rated competencies were Teamwork and Commitment/Engagement although, in general, there appears to be some degree of a ceiling effect, higher at T2, in the results. At the item level, “Collaborates when needed” obtained the highest score (in both pretest and post-test) while the lowest rating was given to the item “Takes occupational risk prevention measures” (in the pretest) and “Has a positive attitude to the changes” (in the posttest) (Table 3 and Table 4).

The scores were higher for women in all competencies: Teamwork (male: 8.15, female: 8.70, t_(24.90)_ = −2.58, *p =* 0.016), Adaptability-Flexibility (male: 7.99, female: 8.55, t_(26.85)_ = −2.59, *p =* 0.015), Commitment/Engagement (male: 8.32, female: 8.75, t_(27.43)_ = −2.86, *p =* 0.008), and Results Orientation (male: 8.23, female: 8.73, t_(25.85)_ = −3.08, *p =* 0.005). Regarding the levels of competency achieved in the post-test, there were significant differences between physicians and nursing assistants, with higher scores for the nursing assistants across all the competencies. Differences obtained among the rest of the groups were nonsignificant.

According to the results of the training intervention, only “Results Orientation” (F_(1.80)_ = 5.941; *p =* 0.017) showed significant improvement in the personnel subsample. For the other competencies, there were no statistically significant changes. Significant improvement was especially important in the case of the items: “Perseveres in attaining the objectives” (t_(80)_ = −2.60, *p =* 0.011) and “Takes occupational risk prevention measures” (t_(80)_ = −4.31, *p <* 0.001). Age correlated positively with the likelihood of modifying behaviors related to “Teamwork” (*r =* 0.25, *p =* 0.025) and “Commitment/Engagement” (*r =* 0.27, *p =* 0.015). Job satisfaction also obtained a positive correlation with the magnitude of the change of “Teamwork” (*r =* 0.23, *p =* 0.045) and “Adaptability-Flexibility” (*r =* 0.25, *p =* 0.02). As for the burnout factors, negative correlations were obtained regarding the magnitude of the change in the four competencies considered, ranging from –0.26 to –0.42 (*p* < 0.01). Negative correlations were also obtained between the degree of competency improvement and the number of attitudinal and aptitude-related complaints, ranging from –0.28 to –0.54 (*p* < 0.01).

Regarding the analysis of discrepancies between the ratings of the three sources, the results presented significant differences in the sample of personnel; in general, supervisors gave the highest scores (Table 5). Finally, it should be mentioned that, for the subsample of supervisors, neither the behavioral change in the analyzed competencies nor the relationships between the magnitude of change and the sociodemographic and organizational variables were statistically significant. It is also important to note that the statistical power permitted by the small sample size (*n* = 13) was 0.19 (under a type-II error rate of 0.81).

## 4. Discussion

This quasi-experimental study aimed to analyze the behavioral change derived from a training intervention, using measurements from two points in time (pretest–post-test) through a 360° MSF model. The competencies studied here are in line with those identified by Donnon et al. [2] and Andrews et al. [18]. It is worth mentioning that most of the studies reviewed in the literature are not homologous to the present study in terms of research design because they either rely exclusively on a cross-sectional approach or analyze the behavioral change but from a qualitative perspective. In this sense, these studies parse the intention to change without providing empirical evidence that the change has ultimately materialized [34,35] or, in other cases, examine self-reported perceptions which, though potentially a useful indicator, provide no evidence that any real change has resulted from the measures [36,37,38]. Additionally, when the literature reports behavioral changes, these are usually related to better relaying information and improved communications [6,7,8,30,35]. Generating multiple feedback that is diverse and relevant to each context is necessary to create a faithful and comprehensive image of one’s self, including one’s strengths and weaknesses [3].

According to the quantitative evidence obtained from the present study, the training intervention had a positive effect on the competency Results Orientation. This improvement has been attributed to the fulfillment of functions, the optimization of resources, and safety. With respect to the magnitude of the change, these results are in line with the longitudinal studies carried out with pediatric residents [17,30,31,32] and similar to those obtained in a sample of family physicians [29] or graduate nursing studies [19]. These limited experiences, which contemplated different time intervals, did not detect significant changes in the set of evaluated competencies. One of the reasons for the minor changes detected could be the ceiling effect, which is reported in most of the analyzed works [6,12,13,23,29,31,32]. A possible explanation for the ceiling effect is that, although it is necessary for the person to be assessed to perceive their raters as credible sources of information [1], choosing them beforehand may produce biased, inflated ratings. Some studies showed that, when raters are not chosen by the person to be evaluated, scores are significantly lower (in other words, these raters tend to be more critical) [23].

In the present study, certain mechanisms were introduced to control or minimize the tendency to rate high: the sample was made up of nonvoluntary subjects, several professional categories were considered, and the raters were not selected by the participant but instead chosen at random according to the analysis of work interactions performed, among other aspects [44]. Even so, one limitation of this study is the impact of the ceiling effect on the results. High ratings could partly explain the slight change produced by not perceiving such change as necessary. Therefore, the assessment process should be maintained over time to familiarize participants with the procedure and thereby minimize the bias.

Our study, following the recommendations of Berk [22], utilized a single questionnaire designed to evaluate different health professions and be completed by all sources, thus allowing for a reduced number of raters. In our opinion, the information obtained through different questionnaires cannot really be considered a 360° MSF assessment sensu stricto.

Although the present study did not include patients due to their tendency to inflate the ratings, the supervisors did not contribute to the reduction of the bias because they gave the highest scores. These results coincide with those obtained in a nursing sample, in which the evaluations of the supervisors were higher than the self-reported ones [45]. A possible explanation, though empirical testing is pending, is that overestimating the team may contribute to a global image of efficient performance and thus avoid an image of poor supervision.

Regarding the feedback provided once the evaluation is completed, it is very similar to that reported by most of the reviewed works, in which a personalized report on the outcomes is provided. As noted in the literature, competency evaluation systems often lack a feedback meeting between supervisor and subordinate. In our case, the supervisors carried out reviews and discussions of the feedback report only when the person evaluated received discrepant ratings. This criterion could help facilitate the sustainability of the system when implementing it on a large scale, though the optimal situation would be for a supervisor to discuss the feedback report with each participant.

Finally, evidence suggests that, when relating behavioral change with sociodemographic and organizational variables, the higher one’s age and job satisfaction, and the lower one’s sense of emotional exhaustion, the more likely it is that a training intervention will lead to improved performance. Furthermore, if such an improvement occurs, it seems to favor a decrease in the number of patient complaints. These results invite managers to consider strategies that increase satisfaction and reduce burnout levels, when implementing a 360° MSF model, in order to promote an improvement in the competencies that impact performance, which can in turn increase the quality of service, and consequently, reduce complaints.

Apart from the stated ceiling effect, the identified main limitations of the study can be the lack of empirical contrast on the invariance of the measurement instruments used for each source [3], and the non-inclusion of external assessment indicators about the target competencies in a complementary way to the MSF model [24].

## 5. Conclusions

Comprehensive feedback using the 360º MSF model can enable health professionals (and even students) to critically evaluate their progress and learning needs and self-identify outcomes. The process can facilitate the increase of confidence in knowledge and skills, and opportunities for behavior change [19]. Although the literature shows an increasing number of studies implementing this model for assessing health professionals’ nontechnical competencies that even develop longitudinal designs, there remains important logistical problems and attitudinal barriers that reduce its optimal functioning—on one hand, the organizational complexity inherent to the appropriate establishment of assessment circuits with raters by different sources, and on the other, the lack of an “evaluative culture” which contribute to encouraging phobias and philias, and corporate attitudes, in general, suppose a cause of rejection by health managers and policymakers, and also health professionals.

To contribute to create and develop that new evaluative culture, this study aims to deepen the experience of implementing a 360° MSF model for the assessment of a training intervention program involving a set of nontechnical competencies of health professionals belonging to a public hospital. Using a longitudinal pretest–post-test design, after the training intervention on the chosen competencies (Teamwork, Adaptability-Flexibility, Commitment-Engagement, Results Orientation, and Leadership Skills), the evidence generated only showed statistically significant improvements of “Results Orientation” competency scores.

The main strengths of this study include the implementation of a training intervention, the rigorous quantitative measurements obtained, and the application of a design with measurements at two points in time. Most studies described in the literature are only qualitative and descriptive, and at most correlational or differential. In spite of the interference of the ceiling effect, it would be interesting to maintain the evaluation model over time because it could contribute to the progressive internalization of an evaluation culture. In this way, biases could be minimized in the interests of a more reliable and accurate assessment, which contributes to improve professional practice and, therefore, increase the quality of patient care.

## Figures and Tables

**Table 1:** Phases and planned actions of the training intervention program and 360° MSF model.

Phase	Actions	Timetable
First year (2016)		
Management committed to the project and selection of target competencies	Overcome internal resistances to achieve commitment and engagement on the part of the management team. Establish strategic guidelines that generate confidence in the process. Gather a panel of experts to select core competencies: Teamwork, Adaptability-Flexibility, Results Orientation and Commitment-Engagement. Determine which services will participate based on established criteria.	February
Protocoldevelopment	Operationalize competencies through behavioral indicators evaluated using a 10-point Likert-type scale. Elaborate two protocols: one for supervisors and the other for professionals.	April–May
Communication plan	Provide health system authorities, hospital management, training department, and trade union reps with information about the model design (objectives, methodology and tool). Inform supervisors and then their teams of the reasons for the evaluation, technical principles, and competencies to be rated. Inform to all the participants in writing about key issues of the process.	June–July
Competencies assessment (pretest)	Analyze labor interactions among professionals to establish feedback circuits. Prepare the protocol (questionnaires: core competencies, satisfaction, and burnout) and deliver the protocol to the participants.	September
Training plan on the development of competencies	Hold training sessions for supervisors (one 5-hour module) and personnel (two 3-hour modules).	October
Pretest data analysis	Analyze discrepancies between assessments provided by different sources. Generate customized reports (scores by items and competencies). Give feedback about discrepant ratings.	November December
Second year (2017)		
Competencies assessment (posttest)	Reassess core competencies following the same methodology established in the pretest.	November
Third year (2018)		
Posttest data analysis	Verify the study hypothesis through statistical analysis.	January February
Feedback about changes	Give personalized reports and hold a discussion with the participants, comparing scores of the Pretest vs. Postest.	March

**Table 2:** Kolmogorov–Smirnov normality tests, and 95% CI of Bliss skewness (g1) and kurtosis (g2) indexes, for the global ratings of all Competencies.

Competence	Kolmogorov Normality Test		[95% CI] Skewness g1		[95% CI] Kurtosis g2	
Timing	T1	T2	T1	T2	T1	T2
Teamwork	0.116 *p* = 0.009	0.132 *p* = 0.001	g1 = −0.59 [−1.12;−0.06]	g1 = −1.93 [−2.47;−1.40]	g2 = −0.25 [−1.31;0.81]	g2 = 5.42 [4.36;6.47]
Adaptability-Flexibility	0.093 *p* = 0.083	0.138 *p* = 0.001	g1 = −0.73 [−1.26;−0.19]	g1 = −1.25 [−1.78;−0.71]	g2 = 0.85 [−0.21;1.91]	g2 = 1.51 [0.45;2.56]
Commitment-Engagement	0.102 *p* = 0.036	0.116 *p* = 0.009	g1 = −0.80 [−1.33;−0.26]	g1 = −0.97 [−1.50;−0.43]	g2 = 0.29 [−0.76;1.35]	g2 = 0.81 [−0.25;1.87]
Results Orientation	0.068 *p* = 0.200	0.121 *p* = 0.005	g1 = −0.21 [−0.75;0.32]	g1 = −0.99 [−1.52;−0.46]	g2 = −0.63 [−1.69;0.43]	g2 = 1.38 [0.32;2.44]
Leadership Skills	0.180 *p* = 0.200	0.160 *p* = 0.200	g1 = −1.23 [−2.46;0.01]	g1 = −0.40 [−1.64;0.83]	g2 = 0.96 [−1.42;3.34]	g2 = −1.09 [−3.47;1.29]

**Table 3:** Ratings of competencies by time point and sources for supervision subsample.

Competence	Average Rating from the Three Sources, M(SD)							
	Self-Assessment		Supervisor		Co-Worker		Total	
Timing	T1	T2	T1	T2	T1	T2	T1	T2
Teamwork	8.24 (0.98)	8.47 (1.03)	8.58 (0.58)	8.77 (0.50)	8.61 (0.75)	8.57 (0.92)	8.48	8.60
Adaptability-Flexi.	8.31 (0.53)	8.57 (0.58)	8.37 (0.97)	7.83 (1.16)	7.89 (1.01)	8.38 (0.92)	8.19	8.26
Commitment-Eng.	8.55 (0.60)	8.65 (0.54)	8.45 (0.54)	8.25 (0.80)	8.31 (0.78)	8.70 (0.60)	8.44	8.53
Results Orientation	8.09 (0.77)	8.49 (0.53)	8.04 (0.94)	7.87 (0.82)	7.99 (0.92)	8.47 (0.70)	8.04	8.28
Leadership Skills	8.47 (0.85)	8.67 (0.50)	8.49 (0.60)	8.19 (0.87)	8.16 (1.10)	8.67 (0.98)	8.37	8.51

**Table 4:** Ratings of competencies by time point and sources for staff subsample.

Competence	Average Rating from the Three Sources, M(SD)							
	Self-Assessment		Supervisor		Co-Worker		Total	
Timing	T1	T2	T1	T2	T1	T2	T1	T2
Teamwork	8.62 (0.88)	8.77 (0.84)	8.70 (1.07)	8.66 (0.78)	8.45 (1.06)	8.25 (1.34)	8.59	8.56
Adaptability-Flexibility	8.09 (1.18)	8.32 (1.04)	8.79 (1.00)	8.64 (0.87)	8.34 (0.93)	8.25 (1.15)	8.41	8.40
Commitment-Engagement	8.34 (0.92)	8.49 (0.90)	8.82 (0.80)	8.88 (0.58)	8.58 (0.86)	8.54 (0.89)	8.58	8.64
Results Orientation **	8.32 (0.80)	8.34 (0.69)	8.21 (0.60)	7.95 (1.21)	7.75 (1.21)	8.37 (1.02)	8.09	8.22

** means *p* < 0.01.

**Table 5:** Simple effects results of the three sources by pretest–post-test for each competency.

Timing	Teamwork	Adaptability Flexibility	Commitment Engagement	Results Orientation
**Pretest** **T1**	Supervisor vs. Self (*t* = 0.61, *p* = 0.546) **Supervisor** vs. Co-workers (*t* = 2.02, *p* = 0.047)	**Supervisor** vs. Self (*t* = 4.28, *p* < 0.001) **Supervisor** vs. Co-workers (*t* = 3.95, *p* < 0.001)	**Supervisor** vs. Self (*t* = 3.82, *p* < 0.001) **Supervisor** vs. Co-workers (*t* = 2.90, *p* = 0.005)	**Supervisor** vs. Self (*t* = 2.89, *p* = 0.005) Self vs. **Co-workers** (*t* = 3.05, *p* = 0.003)
**Posttest** **T2**	**Supervisor** vs. Co-workers (*t* = 2.93, *p* = 0.004) **Self** vs. Co-workers (*t* = 2.96, *p* = 0.004)	**Supervisor** vs. Self (*t* = 2.50, *p* = 0.015) **Supervisor** vs. Co-workers (*t* = 3.24, *p* = 0.002)	**Supervisor** vs. Self (*t* = 3.91, *p* < 0.001) **Supervisor** vs. Co-workers (*t* = 3.64, *p* < 0.001)	**Supervisor** vs. Self (*t* = 2.72, *p* = 0.008) **Supervisor** vs. Co-workers (*t* = 2.01, *p* = 0.048)

The source with statistically significant higher scores appears **in bold.**

## Data Availability

The data presented in this study are available on request from the corresponding author. The data are not publicly available because they belong to the hospital in which the study was developed and cannot be used without clear justification while maintaining strict confidentiality of participants.
